# Efficacy of a Self-Vaccination Strategy for Influenza A Virus, *Mycoplasma hyopneumoniae*, *Erysipelothrix rhusiopathiae*, and *Lawsonia intracellularis* in Swine

**DOI:** 10.3390/vaccines13030229

**Published:** 2025-02-24

**Authors:** Lucas Caua Spetic da Selva, Rebecca Robbins, Courtney Archer, Madelyn Henderson, Jessica Seate, Luis G. Giménez-Lirola, Ronaldo Magtoto, Arlene Garcia, Allen Jimena Martinez Aguiriano, Emerald Julianna Salinas, John J. McGlone

**Affiliations:** 1 Laboratory of Animal Behavior, Physiology and Welfare, Texas Tech University, Lubbock, TX 79409, USA; 2 Pig Improvement Company (PIC), Hendersonville, TN 37075, USA; 3 Department of Animal Science, University of Minnesota, St. Paul, MN 55108, USA; 4 Animal Science Products, Nacogdoches, TX 75961, USA; 5 Department of Veterinary Diagnostic and Production Animal Medicine, College of Veterinary Medicine, Iowa State University, Ames, IA 50011, USA; 6 Laboratory of Animal Health and Welfare, Texas Tech University School of Veterinary Medicine, Amarillo, TX 79106, USA

**Keywords:** pigs, self-vaccination, behavior, environmental enrichment

## Abstract

**Background/Objectives:** Environmental enrichment (EE) devices are required in various countries and markets to promote animal welfare, with dual-purpose devices more likely to encourage adoption. We developed an EE device that allows pigs to self-administer liquids, designed to align with natural and play behaviors, and utilized a maternal pheromone (MP) to attract pigs to the device. This study aimed to evaluate the efficacy of this device in delivering vaccines for Erysipelas, Ileitis, Mycoplasma, and Influenza to growing pigs. **Methods:** Pigs were assigned to three treatments groups: Control (unvaccinated), Hand-Vaccinated (via oral gavage or intramuscular injection), and Self-Vaccinated using the EE device. Baseline samples were collected to determine initial antibody status, and serum and oral fluids’ IgG and IgA levels were measured post-vaccination to assess immune response. Four studies were conducted with 36 pigs (12 per treatment) over a 49-day period. **Results:** Self-vaccination pigs receiving the avirulent live Erysipelas vaccine developed oral and serum antibodies comparable to Hand-Vaccinated pigs. Pigs self-administering the avirulent live *Lawsonia intracelluaris* vaccine developed oral fluid antibodies. In contrast, pigs who received Mycoplasma or Influenza vaccines through self-vaccination exhibited significantly lower antibody levels compared to the Hand-Vaccinated group. **Conclusions:** These findings demonstrated that self-vaccination using EE devices for the oral administration of avirulent live vaccines offers benefits such as reduced labor and improved animal welfare. However, killed vaccines did not elicit sufficient antibody responses, suggesting the need for modified vaccine formulations or administration strategies to improve self-vaccination efficacy.

## 1. Introduction

Bacterial and viral infections pose a threat to pig health, and vaccinations are an important practice to manage infectious diseases [1]. Pigs typically receive at least one injectable vaccination in their lifetime, while replacement breeding stock may receive multiple vaccinations administered by oral or injectable routes. However, the labor-intensive nature of administering injections remains a significant challenge due to a shortage of skilled labor. From 2019 to 2020, employment in hog farming decreased by 0.3% [2]. To address these issues, the agriculture sector must increase mechanization and adopt new technologies to reduce labor requirements while meeting animal needs [3]. Due to the cost of specialized equipment and the need for skilled crews to perform vaccination, both are often utilized between farms in a region and have been implicated in disease spread [4].

Vaccination can be a stressful and potentially painful experience for animals. To mitigate this, needle-free technologies are increasingly being used. These methods offer advantages over traditional needle-based vaccine delivery: they eliminate the risk of broken needles in meat [5] and ensure consistent vaccine delivery [6]. A novel needle-free method of vaccination involves the use of an environmental enrichment (EE) device that pigs use to self-administer vaccines. We recently reported that pigs self-vaccinated against Salmonella and, in doing so, developed oral and serum antibody levels equal to or better than those observed with the hand-administration of the same vaccine. With minimal labor, and on a precise pen basis, pigs can self-administer health products such as vaccines and pheromones [7,8].

*Mycoplasma hyopneumoniae* (*MHP*), which causes enzootic pneumonia, presents a significant respiratory challenge in swine, leading to chronic respiratory issues, poor growth, and increased mortality due to secondary infections [9,10]. Managing *MHP* effectively is critical to reduce disease impact and ensure animal health. Vaccination remains the primary preventive measure; however, the vaccine must be delivered individually to pigs by intramuscular (IM) injection. Thus, improving accessibility and ease of vaccine delivery is essential for managing this pathogen in large-scale operations.

Influenza A virus (IAV) is a zoonotic respiratory virus that causes considerable morbidity, growth impairment, and compromises the immune system, thereby increasing susceptibility to other pathogens [11,12]. IAV vaccines are delivered by individual IM injections which are labor-intensive. Reducing labor through self-administration may encourage the adoption of vaccination. Furthermore, enhancing vaccination efficacy for *Influenza A* is vital to minimizing the spread of respiratory illness within swine populations, other animals, and humans.

*Erysipelothrix rhusiopathiae* (*ERY*), the causative agent of Erysipelas, can result in acute septicemia, skin lesions, and chronic arthritis, negatively impacting productivity and leading to potential carcass condemnation [13,14]. Vaccination is a key strategy to manage *ERY* outbreaks, and providing effective, needle-free vaccine administration options may help reduce both labor requirements and the risks associated with traditional injection methods.

Ileitis, caused by *Lawsonia intracellularis* (*LAW*), leads to proliferative enteropathy, which impairs weight gain and increases mortality [15]. This disease predominantly affects the gastrointestinal tract, resulting in a negative impact on mucosal immunity, which is necessary for effective protection. Vaccination remains a fundamental tool for managing Ileitis. Recently, an injectable bacterin form of the vaccine was developed for precision delivery (Intervet Inc., doing business as Merck Animal Health, a subsidiary of Merck & Co., Inc., Kenilworth, NJ, USA, 2018). This vaccine must be delivered by injection and thus requires more labor compared to its avirulent live oral vaccine counterparts. Therefore, a self-administered vaccine delivery system may provide the precision of an injectable form with the ease of an oral delivery for the control of LAW.

The self-vaccination concept involves pigs self-administering vaccines through direct contact with the EE device. When pigs engage with the device, the vaccine is delivered as a liquid spray and applied to mucosal surfaces, including the nose, eyes, and mouth, facilitating immune stimulation through mucosal exposure. This study aimed to evaluate the efficacy of the EE sprayer in delivering vaccines targeting MHP, IAV ERY, and LAW. We hypothesized that self-administration through the EE sprayer could elicit sufficient immunological responses, comparable to the traditional hand-vaccine delivery method. To assess the efficacy of the delivery method, we collected serum and oral fluid samples to measure antibody development for each antigen following vaccine administration.

## 2. Materials and Methods

### 2.1. General Information

The protocol was approved by the Texas Tech University (TTU) Institutional Animal Care and Use Committee (IACUC # 2023-1347, approved 19 December 2023) prior to the start of the work. For each vaccine trial, 36 growing pigs, with an average of 20 weeks of age, were randomly selected from a larger population (Camborough sow x PIC^®^ 800 boar). Pigs had access to a corn–soy-based diet mixed at the TTU Swine Unit formulated to meet or exceed NRC (National Research Council) nutrient requirements for swine. The flooring was concrete slats over an under-floor flushing system. Air temperature and ventilation were consistent with the Guide for the Care and Use of Agricultural Animals [16]. The experimental finishing barn was a model barn, and similar to barns on commercial farms. Pigs had approximately 1.9 m2 of floor space per pig. Four pigs were housed per pen. Each pen had a self-feeder, and an adjustable-height nipple mounted to the wall for water delivery. Feed and water were provided ad libitum. Farm workers did not enter the test pens. When researchers entered the experimental pens, they wore disposable plastic shoe covers and always entered the Control (non-vaccinated pigs) pens before the treatment pens. All serum and oral fluids were kept frozen until study completion, then shipped to the Iowa State University Veterinary Diagnostic Laboratory (ISU-VDL; Ames, IA, USA). The laboratory was blind to the treatment of the samples.

### 2.2. Maternal Pheromone (MP) Pilot Study

Once thawed, frozen live attenuated vaccines needed to be consumed within 6 h [17]. This new concept required pigs to be attracted to the self-sprayer so they could receive the vaccine while it was still viable. A pilot study was conducted to evaluate the effectiveness of an MP in attracting pigs to the EE device. Twelve finishing pigs were divided into three pens and would be used later as a part of Trials C and D (below). The experiment ran for two consecutive days; one day an EE device was used without MP and the next day with MP. To confirm vaccine delivery, the EE device dispensed water mixed with non-toxic food dye. The dye marked the pigs, allowing the visual confirmation of contact with the device, serving as an indicator of successful vaccine delivery. Video recordings were evaluated to track the time to first spray and the frequency of each pig’s interactions with the EE device. Data from the two days were compared using a paired *t*-test, with significance set at *p* < 0.05. The normality of the differences between paired observations was assessed using the Shapiro–Wilk test and verified through a Quantile–Quantile (Q-Q) plot. The primary question was whether the MP would encourage a greater use of the EE device compared to a control EE device and whether all the pigs would receive a spray before the vaccines’ viability expired (approximately 6 h). The statistical power of the paired *t*-test was evaluated to determine the likelihood of detecting true differences between groups. Power analysis was performed based on the sample size of 12 paired observations, an assumed effect size of 0.85, and a two-sided significance level of 0.05. This approach ensured that the study design was adequately powered to detect meaningful differences between treatment groups.

### 2.3. Experimental Design of Vaccine Studies

Four vaccine efficacy studies were conducted using two batches of finishing pigs (Table 1). At the time of these studies, the herd was naïve for porcine reproductive and respiratory syndrome virus (PRRSV), porcine epidemic diarrhea virus (PEDV), porcine delta coronavirus (PDCoV), transmissible gastroenteritis virus (TGEV), and *MHP*. No *MHP*, IAV, or *LAW* vaccines were currently being used in the herd; however, some pigs had background titers to *LAW* prior to the study. All pigs had received *ERY* bacterin at 21 days of age. Three pens of pigs were assigned to each treatment; in the *MHP* and IAV trial (Trial A and B, respectively), each pen contained 2 castrated males and 2 females, and in the *ERY* and *LAW* (Trial C and D, respectively) trial, each pen contained 3 castrated males and 1 female. All pens were in the same barn. Each treatment group was accommodated in blocks of three adjacent pens, with contact possible through fencing within those treatment groups only. An empty pen, not containing pigs, was placed between treatment blocks to prevent physical contact between treatment groups. The pen configuration was to ensure no inadvertent exposure occurred between controls and vaccinated pigs or between vaccination routes. Treatments and measures were applied to the pig; therefore, the pig was the experimental unit. Our previous work demonstrated that 12 pigs per treatment was sufficient to detect meaningful differences in antibody levels [7]. Sampling days for each trial are given in Table 1.

**Figure 1:**
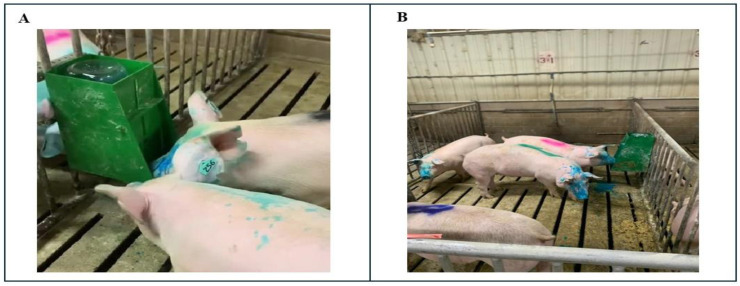
(**A**) Example of a pig self-vaccinating using the EE device. (**B**) Pigs exposed to EE device. The blue dye was added to the vaccine to identify that the pig had been exposed to the vaccine.

Pigs were randomly assigned to one of three treatment groups: (1) Control pigs that received no vaccine or exposure to the EE device; (2) Hand-Vaccinated pigs in Trials A and B that received IM injections of commercially licensed vaccines, while those in trial C and D received vaccines delivered orally by hand with a syringe; and (3) Self-Vaccinated pigs were exposed to an EE device attached to each pen, which they could activate by pressing a panel with their snouts. Upon activation, the device delivered a spray (4 mL per activation) directed toward the pig’s facial region, ensuring contact with the nares, eyes, and mouth. The device was designed to maintain consistent vaccines by controlling spray volume, droplet size, and spray angle, providing uniform exposure regardless of individual pig behavior (Figure 1). At the time of vaccine administration, 15 mL of MP in corn oil [18] was sprayed on the front panel of the EE device to encourage activity with the device. Cameras had an overhead view and Network Video Recorders (NVRs) were used to record the Self-Vaccinated group to determine if all pigs received the vaccine. Sample collection was conducted at specific intervals post-vaccination (Table 1) to monitor antibody responses in the pigs.

### 2.4. Vaccines

Commercially available vaccines licensed by the USDA for delivery in combination were selected for the two trials. Vaccines were handled according to the label instructions. The pigs in the Hand-Vaccinated groups each received a full dose of the vaccine as described on the label, and it was delivered orally (*LAW* and *ERY*) or IM in the neck (*MHP* and IAV) in a single administration.

In Trial A, *MHP* bacterin was added to a lyophilized, multivalent killed IAV vaccine (FluSure XP/RespiSure-One, Zoetis Inc., Kalamazoo, MI, USA), and 2 mL was delivered IM by a 16G 1” detectable needle (Neogen, Ideal D3, Lansing, MI, USA) on day 0 to each pig in the Hand-Vaccinated group. In accordance with the label, a 2 mL dose of the IAV vaccine (FluSureXP) was given IM 21 days later to Hand-Vaccinated pigs. For delivery in the EE device, the same 2 mL volume of the *MHP*/IAV vaccine per pig was added to 192 mL of water containing sodium thiosulfate with non-toxic blue dye (Reload Pack), prepared at a ratio of 1 bottle of non-toxic blue dye to 3.8 L water. Self-Vaccinated groups were also exposed to a 2 mL dose of the Influenza A vaccine 21 days after the first administration via the same route.

In Trial C and D, frozen avirulent live *ERY* and *LAW* vaccine cultures (Ingelvac Ery-ALC and Enterisol Ileitis FF, Boehringer Ingelheim Animal Health, Duluth, GA, USA) were thawed in a cold water bath prior to mixing with water containing a vaccine stabilizer that contains antioxidant, multiple buffers, electrolytes and a non-toxic blue dye as described in the manufacturer’s product insert (Vac-Pac^®^, Animal Science Products, Inc., Nacogdoches, TX, USA). Each pig in both the Hand-Vaccinated and Self-Vaccinated groups received 1.25 doses of LAW at a volume of 1.25 mL and 1.25 doses of ERY at a volume of 0.5 mL. The volume of LAW and ERY vaccines were combined in 5.25 mL of Vac-Pac^®^ treated water and a total 7 mL volume was delivered orally by syringe to each individual pig. 

### 2.5. Sample Collection

All research personnel entering the pens wore PPE including coveralls, boots, boot covers, and latex gloves. Shoe covers were changed between treatment groups. Individual oral fluid samples were collected using cotton balls tied to a string. Individual pigs were allowed to chew on cotton balls until moistened for each occurrence of oral fluid sample collection (Trials A and B at −7, 21, 28, and 45 DPV, and Trials C and D at 0, 21, 28, 42, and 45 DPV).

Group-based oral fluids were collected from each pen using 1.6 cm diameter, unbleached cotton rope. Oral fluids were tested by RT-qPCR for IAV at days 0 and 21 in Trial B and for *LAW* at days 0 and 49 in Trial D at the ISU veterinary diagnostic laboratory (VDL).

Blood samples were collected from the vena cava by venipuncture. For blood collection, pigs were restrained using a snare as appropriate for their production age. Blood samples were centrifuged at 4000× *g* for 5 min. Serum was transferred to snap-cap safe-lock microcentrifuge tubes. Serum and individual oral fluid samples from each trial were labeled in a blind manner to those performing the assays, stored at −20 °C, and shipped to ISU-VDL frozen for analysis.

### 2.6. Serology Methods

Antibody levels were determined using previously optimized ELISAs. Serology was performed to measure IgG, IgA, and/or total antibodies in serum and oral fluids. The ISU-VDL was blind to treatments for the samples they assayed.

#### 2.6.1. Equipment and General Protocol for ELISAs

All ELISAs were conducted using an ELx405 plate washer (Biotek Instruments Inc., Winooski, VT, USA) for washes at 300 µL per well, an EMax Plus microplate reader (Molecular Devices, San Jose, CA, USA), and SoftMax Pro 7.0 software. Reagents, including diluted or undiluted samples and controls, conjugates, substrates, and stop solutions, were dispensed at a volume of 100 µL per well. The validity of each assay was confirmed for every plate using internal, commercial or in-house, positive and negative controls.

#### 2.6.2. Influenza A Virus ELISAs

For the commercial blocking ELISA, a Swine Influenza Virus Antibody kit (Ref# 99-0000900; Idexx, Westbrook, ME, USA) was utilized following the manufacturer’s instructions. Samples were diluted 1:10 and added to antigen-coated plates along with undiluted positive and negative controls. The plates were incubated at room temperature (RT; 22–24 °C) for 60 min and subsequently washed four times using the Microplate Washer. After washing, conjugate was added to each well and incubated for an additional 30 min at RT. Following another four washes, substrate was added and incubated for 15 min at RT in the dark. The reaction was terminated with the stop solution, and absorbance was measured immediately at 650 nm. Sample-to-negative (S/N) ratios were calculated. The S/N ratio was calculated as (OD of sample − OD of negative control)/(OD of positive control − OD of negative control), which is commonly used in competitive ELISAs where a lower OD indicates higher antibody reactivity.

In the in-house IgA ELISA adapted from [19], Maxisorp ELISA plates (Thermo Scientific, Roskilde, Denmark) were coated with 1.2 µg of nucleoprotein (NP) antigen (1:1000 dilution from a 1.2 mg/mL stock) in phosphate-buffered saline (PBS; Gibco^®^; Life Technologies Corp., Grand Island, NY, USA) at a pH of 7.4 and incubated for 16 h at 25 °C. After four washes, plates were blocked with 1% bovine serum albumin (Jackson ImmunoResearch, West Grove, PA, USA) at 300 µL per well for 2 h at RT, dried for 3 h at 37 °C, and stored at 4 °C with desiccant packs. For the assays, 100 µL of 1:1 diluted oral fluid samples and controls were added to the plates and incubated for 60 min at 37 °C. The plates were washed four times, and HRP-conjugated goat anti-pig IgA (Bethyl/Fortis Life Sciences, Montgomery, TX, USA) at a dilution of 1:3000 was added to all wells and incubated for 60 min at 37 °C. Following four additional washes, BioFX TMB One Component HRP Microwell Substrate (Surmodics IVD, Inc., Eden Prairie, MN, USA) was added and incubated for 5 min at RT in the dark. The reaction was stopped with 100 µL of BioFX 450 nm Liquid Nova-Stop Solution (Surmodics), and plates were read immediately in the microplate reader at a wavelength of 450 nm.

The commercial blocking ELISA for IAV was performed according to the manufacturer’s instructions, with absorbance measured at 650 nm due to the absence of the acidic stop solution, which preserves the blue color of the TMB reaction. In contrast, the in-house ELISA adapted for IgA detection used an acidic stop solution, resulting in a yellow endpoint measured at 450 nm, following standard TMB assay protocols.

#### 2.6.3. *Mycoplasma hyopneumoniae* ELISAs

The commercial serum IgG ELISA for *M. hyopneumoniae* (Ref# 99-06733; Idexx, Westbrook, ME, USA) was utilized according to the manufacturer’s recommendations. Serum samples were prediluted to 1:40 in sample diluent and added to antigen-coated plates along with undiluted positive and negative controls. The plates were incubated at RT for 30 min and then washed four times using a Microplate Washer. Conjugate was added to each well and incubated for an additional 30 min at RT. After four more washes, the substrate was added and incubated for 15 min at RT in the dark. The reaction was halted with the “stop” solution, and absorbance was measured at 650 nm. Results were expressed as sample-to-positive (S/P) ratios. The S/P ratio was calculated as (OD of sample − OD of negative control)/(OD of positive control − OD of negative control), a standard approach in indirect ELISAs to normalize sample reactivity against known positive and negative controls.

For the adapted oral fluid IgG ELISA, the Idexx *M. hyopneumoniae* ELISA kit was modified to assess IgG in oral fluid samples. The kit conjugate was substituted with goat anti-pig IgA HRP conjugate (Bethyl/Fortis Life Sciences), the substrate was replaced with BioFX TMB One Component HRP Microwell Substrate (Surmodics), and the stop solution was changed to BioFX 450 nm Liquid Nova-Stop Solution (Surmodics). Additionally, new internal positive and negative oral fluid controls with known M. hyopneumoniae status were included in each run. Samples were diluted 1:1, added to the plates, incubated at 4 °C for 16 h, and washed four times. The kit conjugate was replaced by HRP-conjugated goat anti-pig IgG (Fc) (Bethyl/Fortis Life Sciences), diluted 1:3500 in conjugate stabilizer, and incubated for 30 min at 37 °C. After four additional washes, BioFX TMB One Component HRP Microwell Substrate (Surmodics) was added and incubated for 5 min at RT in the dark. The reaction was stopped by adding BioFX 450 nm Liquid Nova-Stop Solution (Surmodics), and absorbance was measured at 450 nm.

#### 2.6.4. LAW Intracellularis ELISAs

For the commercial blocking ELISA, antibodies against *L. intracellularis* in serum were assessed using a blocking ELISA kit (Ref# SV-122275; Svanova/Indical Bioscience GmbH, Leipzig, Germany) following the manufacturer’s instructions. Briefly, serum samples and kit internal controls were diluted 1:10 and dispensed into antigen-coated microplates. Plates were incubated at 37 °C for 60 min to allow specific antibodies in the samples to bind to the coated antigen. Following three washes with microplate washer to remove unbound antibodies, the kit’s enzyme-conjugated detection antibody was added to each well and incubated for an additional 60 min at 37 °C. After three additional washes, the substrate was added and incubated for 10 min at room temperature in the dark. The reaction was terminated by the addition of the stop solution provided in the kit, and absorbance was measured at 450 nm using a microplate reader. Results were calculated as percentage inhibition (PI), following the kit’s specification.

Adaptations for serum and oral fluid IgA ELISAs involved modifying the Svanova/Indical *L. intracellularis* antibody ELISA into an indirect format. For serum samples, a dilution of 1:100 with kit diluent was prepared and dispensed into antigen-coated plates, which were incubated at room temperature for 60 min and washed four times. HRP-conjugated anti-pig IgA (Bethyl/Fortis) was added at a dilution of 1:3000 and incubated for another 60 min at room temperature. Following four additional washes, kit substrate was added, and the plates were incubated for 5 min at room temperature in the dark. The reaction was terminated with the kit stop solution, and absorbance was measured at 450 nm.

For oral fluid samples, diluted 1:1 kit samples were dispensed into antigen-coated plates and incubated for 16 h at 4 °C. After washing four times, 100 µL of HRP-conjugated goat anti-pig IgA was added at a dilution of 1:6000 and incubated for 60 min at room temperature. The plates were washed again, substrate was added, and incubation proceeded for 5 min at room temperature in the dark. The reaction was stopped with the stop solution, and absorbance was measured at 450 nm.

#### 2.6.5. *Erysipelothrix rhusiopathiae* FMIA

The testing of serum and oral fluid samples for IgG antibodies against *E. rhusiopathiae* was conducted using a fluorescent microbead immunoassay (FMIA) based on an immunogenic recombinant polypeptide (rSpaA415) derived from the SpaA protein, following previously described methods [20,21,22]. Antigen-coupled magnetic microbeads were sonicated and vortexed for 30 s, then diluted in bead buffer (StabilGuard Stabilizer; Surmodics) to a concentration of 2500 beads per well (50 beads/µL). Serum samples were diluted 1:50 in 10% goat-serum-based assay buffer, while undiluted oral fluid samples (50 µL) were mixed with 50 µL of the bead suspension to achieve a final reaction volume of 100 µL. Bio-Plex Pro flat-bottom plates (Bio-Rad Laboratories Inc., Hercules, CA, USA) were sealed and incubated at RT between 1 h (serum) and 2 h (oral fluids) with shaking at 650 rpm, followed by three washes with PBS containing 0.1% Tween 20 (PBST). Biotinylated goat anti-pig IgG (Fc) was added at a dilution of 1:1000 (50 µL per well) and incubated for 30 min at RT with shaking. After washing, streptavidin phycoerythrin conjugate (Moss Inc., Pasadena, MD, USA) was added at a dilution of 1:100 in assay buffer (50 µL per well) and incubated for another 30 min at RT with shaking. Following a final series of washes, 100 µL of assay buffer was added, and the plates were shaken for 5 min before being read on the Luminex 200 instrument (Luminex^®^/DiaSorin, Austin, TX, USA). Median fluorescence intensity (MFI) was measured and corrected for background levels by subtracting the negative antigen signal from the positive antigen signal. The results were expressed as S/P ratios.

### 2.7. Statistical Analyses

Statistical analyses were performed using SAS v9.4 (SAS Institute Inc., Cary, NC, USA), employing mixed-effect models with the pig as the experimental unit, the random effect of the days post-vaccination, and treatment groups as fixed effects, to assess serology as the primary response. The analyses were conducted using PROC MIXED, with the Tukey–Kramer post hoc test applied to compare groups across the days. The validity of the test was evaluated by assessing the homogeneity of variance and the normality of residuals through the inspection of a residual plot. Animals with measured titers over three standard deviations above the overall mean on day 0 were considered outliers and excluded from further analysis to maintain data integrity (one pig was excluded from each trial in A and B). Data transformation was performed in the case of severe violations of normality by adding a constant to the antibody titers and then performing a log transformation. The transformation used the formula log_10_(Antibody titers + a), where *a* is a constant calculated as 1 minus the minimum antibody titer value (a = 1 − min(antibody titer)). This ensured that all titers were non-negative, allowing for the application of logarithmic transformation. The key response criteria were whether the Self-Vaccinated pigs were similar to or different from the Hand-Vaccinated or no vaccine Control groups at each time point of blood or oral fluid collection. If Self-Vaccinated pigs developed antibody levels similar to control (non-vaccinated) pigs, then self-vaccination did not work using the vaccines tested. If Self-Vaccinated pigs achieved antibody levels equal to Hand-Vaccinated pigs, then self-vaccination is an acceptable alternative to labor-intensive individual vaccinations.

## 3. Results

### 3.1. Pilot Study with MP

**Figure 2:**
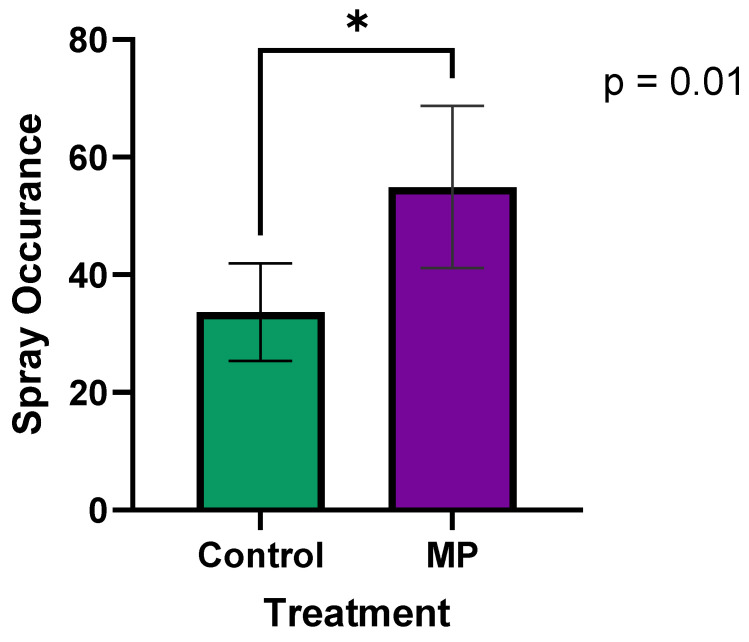
The effect of MP on spray occurrence (total sprays over 5 h) pig interaction with the EE device. The frequency of spray occurrence was significantly (*p* < 0.05) higher in the MP treatment group compared to Control. Data were collected over 24 h periods: one day without MP (Control), and another day with MP. An asterisk (*) indicates a significant difference (*p* < 0.05).

A paired *t*-test was performed to compare the occurrence of the pigs spraying themselves between Control and MP treatment groups. The results, shown in Figure 2, indicate that pigs in the MP group had significantly higher spray interactions (mean = 54.9) compared to the Control group (mean = 33.7; *p* = 0.01). All pigs were exposed to the liquid in the sprayer when the MP was present (with a range of exposure times from 5 s to 160 min), and pigs sprayed themselves the first time an average of 20.2 ± 12.4 min after the MP was applied, which was significantly faster than in the Control group without MP (49.9 ± 15.1 min). This suggests that the use of MP effectively attracted pigs to the sprayer, leading to more frequent interactions that were reliable (all pigs were sprayed in a timely manner). This finding also confirms that pigs will expose themselves to vaccines well within the optimal window to maintain the viability of an avirulent live vaccine.

To ensure the viability of the paired *t*-test, the normality of the differences between paired observations was assessed using the Shapiro–Wilk test (*p* = 0.996) and confirmed with a Q-Q plot, indicating that the data met the normality assumption. The statistical power of a significance level of 0.05 (two-sided) resulted in a power of 0.77 (77%). This indicates that there is a 77% chance of correctly detecting a true difference between groups, which is considered reasonably strong due to the large observed effect size.

### 3.2. Trial A: Mycoplasma hyopneumoniae

**Figure 3:**
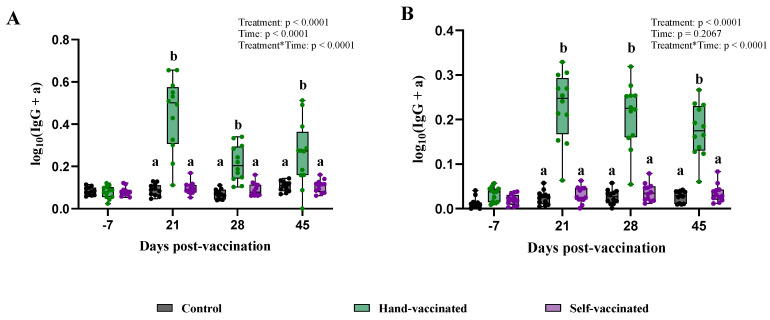
Time course of IgG antibody (data transformed) development for Trial A—*MHP*. Control group received no vaccine, Hand-Vaccinated received IM, and Self-Vaccinated group used the EE device. (**A**)—Serum IgG levels and (**B**)—oral fluid IgG levels. Box-plot illustrates the distribution of IgG levels across the groups. Different letters (a, b) indicate statistically significant differences between groups at each time point (Tukey–Kramer post hoc test, *p* < 0.05).

The data violated the equal variance among treatments assumption, therefore requiring data transformation (for both serum and oral fluids). Results from day −7 showed that all pigs from the three treatments had low S/P values (IgG) in both serum and oral fluids; detailed titer information from Trial 1 can be found in Table 2. At 21 DPV, an increase in IgG levels in serum and oral fluids was observed for the Hand-Vaccinated group, and these levels remained constant throughout the duration of the study. In contrast, IgG levels measured in samples from the Control and Self-Vaccinated groups remained stable throughout the experiment, with no significant changes detected (Figure 3). The mixed-effect model revealed treatment effects (*p* < 0.0001, for serum and oral fluids); however, the differences were between the Hand-Vaccinated and Control treatment groups.

### 3.3. Trial B: Influenza A Virus

**Figure 4:**
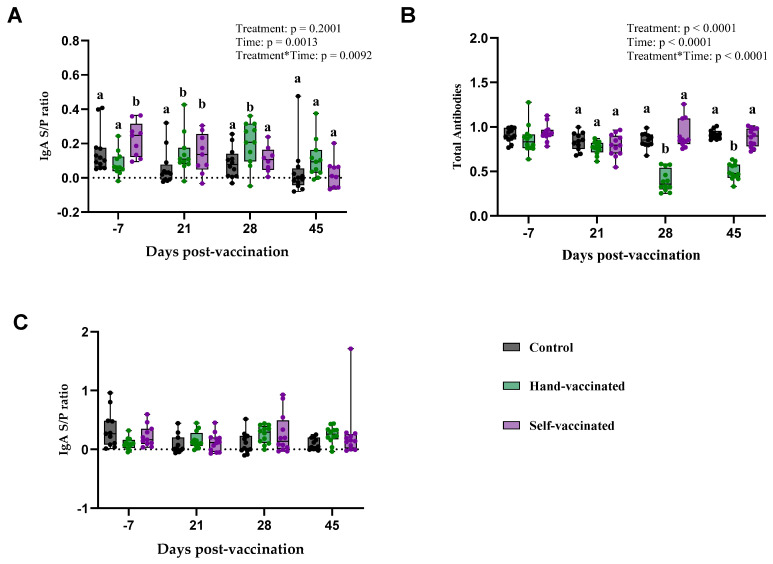
Time course of IgG antibody development for Trial B—IAV. Control group received no vaccine, Hand-Vaccinated received IM, and Self-Vaccinated group used the EE device. (**A**)—Serum IgA levels, (**B**)—serum TAB, and (**C**)—oral fluid IgA. Box-plot illustrates the distribution of IgG levels across the groups. Different letters (a, b) indicate statistically significant differences between groups at each time point (Tukey–Kramer post hoc test, *p* < 0.05).

The key findings of serum and oral fluid antibody titers (IgA and TAB) are summarized in Table 3 and Figure 4.

The serum IgA response to the Influenza vaccine is presented (Panel A, Figure 4). There was no significant effect of treatment (*p* = 0.20) on any antibody measures. However, a significant effect of time (*p* = 0.0013) and treatment by time (*p* = 0.0092) were observed. Specifically, on day 28 and 45 post-vaccination, pigs in the Hand-Vaccinated group demonstrated higher IgA levels compared to the Control and Self-Vaccinated groups. By day 45, IgA levels in all groups had decreased, with no significant differences among the three groups.

Serum total antibody levels (Panel B—Figure 4) showed a significant effect treatment (*p* < 0.0001), time (*p* < 0.0001), and the treatment-by-time interaction (*p* < 0.0001). TAB values measured in samples from Control and Self-Vaccinated groups were significantly lower compared with those from the Hand-Vaccinated group. Because this is an inverse measure, the lower values in the Hand-Vaccinated group indicate a higher concentration of antibodies. Notably, antibody levels measured in the Hand-Vaccinated group peaked on days 28 and 45, occurring seven days after the booster, indicating that the booster successfully stimulated strong and sustained antibody production during these periods among Hand-Vaccinated pigs.

Oral fluid IgA responses (Panel C—Figure 4) showed no significant effects of treatment, time, and treatment-by-time interaction. The IgA levels in oral fluids remained consistent across all time points and treatment groups, indicating that the vaccine delivery method may have a stronger impact on serum IgA than on mucosal IgA levels in oral fluids. Self-vaccination with this antigen did not generate serum or oral fluid antibodies equivalent to those in Hand-Vaccinated pigs. Self-Vaccinated pig titers did not differ from the Controls’ (non-vaccinated) pig titers.

### 3.4. Trial C: Erysipelothrix rhusiopathiae

IgG oral fluids and serum levels measured on day 0 were low across all three groups. Antibody concentrations for *ERY* are shown in Table 4.

**Figure 5:**
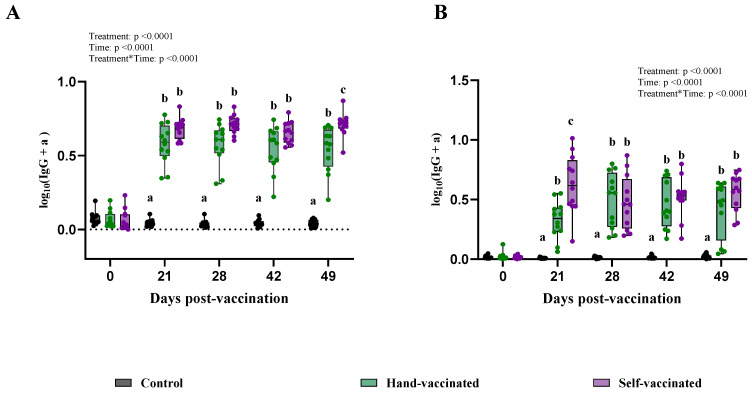
Time course of IgG antibody (data transformed—serum) development for Trial C—*ERY*. Control group received no vaccine, Hand-Vaccinated received IM, and Self-Vaccinated group used the EE device. (**A**)—serum IgG levels and (**B**)—oral fluid IgG levels. Box-plot illustrates the distribution of IgG levels across the groups. Different letters (a, b, and c) indicate statistically significant differences between groups at each time point (Tukey–Kramer post hoc test, *p* < 0.05).

Analyses of serum IgG showed significant effects for treatment, time, and interaction by treatment by time (*p* < 0.0001 for all three). During days 21, 28, and 42, antibody levels measured in samples from the Hand-Vaccinated and Self-Vaccinated groups were not significantly different. However, on day 49, samples from the Self-Vaccinated group showed higher antibody levels than those from the Hand-Vaccinated group (Panel A—Figure 5).

For oral fluid IgG, significant differences were found for treatment, time, and the treatment-by-time interaction (*p* < 0.0001 for all three). At 21 DPV, the Self-Vaccinated group showed a better seroconversion than the Hand-Vaccinated group (Panel B—Figure 5), suggestive of a more robust early immune response for mucosal protection. On days 28, 42, and 49, their antibody levels between these two groups did not differ. For this vaccine, the Hand-Vaccinated and self-administered vaccine groups had similar antibody responses.

### 3.5. Trial D: Lawsonia intracellularis

Key information on antibody concentrations for pigs exposed to the *LAW* vaccine is given in Table 5. Serum IgA levels measured in collected samples did not show significant differences for treatment and the treatment-by-time interaction (*p* = 0.867 and *p* = 0.816, respectively). However, an expected time effect was observed (*p* < 0.0001). Serum TAB levels also did not show statistical significance for treatment (*p* = 0.178) or the treatment-by-time interaction (*p* = 0.308), but significant changes were observed over time (*p* < 0.0001).

**Figure 6:**
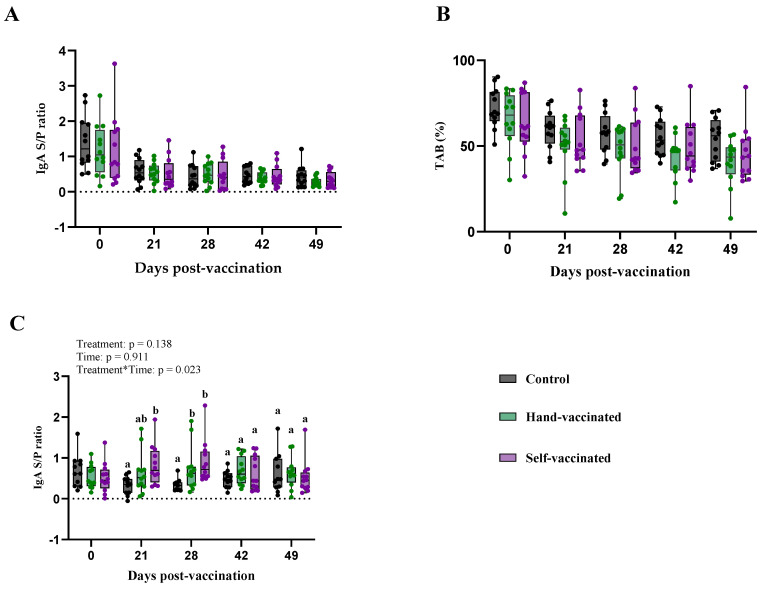
Time course of IgG antibody development for Trial D, *LAW*. Control group received no vaccine, Hand-Vaccinated group received IM, and Self-Vaccinated group used the EE device. (**A**)—Serum IgA levels, (**B**)—serum TAB, and (**C**)—oral fluid IgA. Box-plot illustrates the distribution of IgG levels across the groups. Different letters (a, b) indicate statistically significant differences between groups at each time point (Tukey–Kramer post hoc test, *p* < 0.05).

For IgA in oral fluid samples collected on day zero, antibody levels for LAW were already present across the three treatment groups, as shown in Panel C, Figure 6. The treatment-by-time interaction was statistically significant (*p* = 0.023), whereas neither treatment (*p* = 0.14) nor time (*p* = 0.91) alone showed significant effects. On day 21, samples from the Self-Vaccinated treatment group had higher mucosal antibody titers than those from the Hand-Vaccinated group, but by day 28, there was no longer a significant difference between the two groups. After day 28 of post vaccination, a decrease in the immune response was observed for both the Hand-Vaccinated and Self-Vaccinated groups resulting in all treatments being statistically equal (Figure 6).

## 4. Discussion

This study evaluated the efficacy of a novel vaccination strategy using an EE sprayer for swine pathogens, including *MHP*, IAV, *ERY*, and *LAW*. While the self-vaccination protocol previously demonstrated efficacy for Salmonella, this study extends its successful application to *LAW* and *ERY*. However, the protocol did not elicit a meaningful antibody response when the dose specified on the label of a commercially licensed and approved killed IAV-S or *MHP* vaccine was self-administered by growing pigs.

One of the key advantages of non-invasive vaccination methods, such as the EE sprayer, is the reduction in handling-related stress in pigs. Reduced stress has been associated with improved immune function and lower susceptibility to infections, which may indirectly enhance overall herd health. This stress reduction is an important welfare consideration, as traditional vaccination methods often involve restraint and handling, which can trigger stress-induced immunosuppression [23].

Our pilot study confirmed that incorporating MP significantly increased pigs’ interaction with the EE sprayer, resulting in a higher frequency of spray events compared to Controls. MP, mimicking natural chemical signals, promoted engagement with the EE device and ensured timely self-vaccination before vaccine viability deteriorated. Recorded video data confirmed that all pigs were sprayed at least once within 90 min, consistent with findings that MP expedites engagement and ensures the utility of live vaccines having limited viability in field conditions [18,24].

### 4.1. MHP Vaccine

Seroconversion for systemic and mucosal IgG responses was detected in samples from the Hand-Vaccinated group, whereas no seroconversion was observed in samples from the Self-Vaccinated and Control groups. The lack of response in the Self-Vaccinated group may be attributed to the use of a killed vaccine (RespiSure-ONE, Zoetis) and the novel delivery route, which might not have provided sufficient antigen exposure. The efficacy of inactivated vaccines often depends on precise dosing and the use of adjuvants, which are challenging to achieve in self-administration formats. Previous studies have highlighted variability in immune responses to *MHP* vaccines, influenced by genetic and environmental factors, as well as the poorly understood mechanisms of immunity against *MHP* [25,26].

In contrast, live attenuated vaccines and avirulent live cultures may be better suited for self-vaccination. These vaccines stimulate a comprehensive immune response, including both systemic and mucosal immunity, which is particularly advantageous for respiratory pathogens like *MHP* [27,28,29,30]. With delivery through the EE device, live vaccines could benefit from the natural attraction induced by MP, increasing the likelihood of effective delivery and uptake. However, such *MHP* vaccines are currently licensed only in specific countries, such as Mexico and China [31].

### 4.2. IAV-S Vaccine

TAB levels measured in samples from the Hand-Vaccinated group were significantly higher on day 28 (seven days after the booster vaccination), while the mucosal IgA levels measured in samples from both Self- and Hand-Vaccinated groups remained negligible. This indicates that the observed increase in TAB reflects systemic antibody production beyond IgA, likely involving other immunoglobulin classes. These findings align with the typical systemic immunity induced by killed vaccines, which often fail to elicit strong mucosal responses [32,33,34,35]. Like the *MHP* trial, this highlights the limitation of the EE device in effectively delivering killed vaccines.

Interestingly, recent research demonstrated that live attenuated and replicon-based Influenza vaccines provided significantly improved protection in piglets compared to commercial inactivated vaccines. These novel vaccine platforms not only reduced viral replication but also elicited stronger mucosal and systemic immune responses, making them potentially more suitable for self-vaccination approaches. This is particularly relevant when using devices like the EE sprayer, as these vaccines rely less on precise dosing and adjuvant use for efficacy [36].

### 4.3. ERY Vaccine

Samples from the Self-Vaccinated group showed an early mucosal immune response compared to those from Hand-Vaccinated group, underscoring the potential advantages of self-vaccination for live vaccines [32]. Live attenuated vaccines, capable of limited replication, mimic natural infections and stimulate both mucosal and systemic immunity [35]. Traditional administration methods, such as hand-drenching or water-line delivery, are labor intensive and inconsistent [27]. The EE sprayer offers a more efficient alternative, delivering consistent vaccine doses and enhancing uptake through multiple mucosal surfaces, as observed in our earlier study with the Salmonella vaccine [7].

### 4.4. LAW Vaccine

Early mucosal IgA response was detected in oral fluid samples from the Self-Vaccinated group, highlighting the role of mucosal immunity in protection against gastrointestinal pathogens [37]. Systemic responses were minimal, consistent with the localized protection conferred by mucosal vaccines. Oral vaccines, such as those used for *LAW*, are particularly effective for self-administration as they stimulate mucosal immunity, critical for enteric pathogens [38]. These findings align with previous studies reporting stronger mucosal responses to *LAW* exposure and reinforce the relevance of oral IgA for enteric diseases [38,39,40,41].

### 4.5. Limitations and Future Directions

This study was conducted under controlled conditions, which may not fully replicate the environmental variability of commercial swine farms. Field studies are needed to evaluate the EE device’s efficacy in larger populations and determine the optimal pig-to-sprayer ratio for live vaccine viability within the six-hour window. Additionally, the adaptation of commercial ELISA kits into an indirect format for detecting IgA in oral fluids represents a novel advancement. To our knowledge, this is the first report of such a modification, enabling the sensitive and cost-effective surveillance of large populations. Further validation of these assays will be crucial to confirm their reliability and expand their application in field studies.

The findings suggest that the self-administration of killed vaccines, for IAV-S and *MHP*, may not be effective when delivered via the EE device. Inactivated vaccines typically rely on systemic immunity and often require adjuvants or higher doses to enhance efficacy. These characteristics pose challenges for self-vaccination formats without precise dose controls [42,43,44]. These challenges could be overcome with subsequent research.

Moreover, the absence of a pronounced immune response in Self-Vaccinated pigs may not reflect vaccine inefficacy but could also indicate a less debilitating immune reaction. Non-invasive methods might induce more balanced immune responses that minimize systemic inflammation [45], potentially benefiting the animal’s overall well-being while still providing protection. Further studies are needed to evaluate the qualitative aspects of these immune responses.

The study goal was to demonstrate innovation in vaccine delivery; only vaccines commercially licensed, available, and accessible in the United States at the time of the study were used. The *M. hyopneumoniae* and *IAV-S* vaccine used in Trial A and B are commercially licensed, killed whole inactivated (i.e., killed) vaccines. Killed vaccines may not produce robust cellular or mucosal immunity [46]. Therefore, vaccine type could be a reason for a lack of antibody response following the delivery of the killed *IAV-S* vaccine with self-sprayer. Based on prior findings [7] and work herein, a live attenuated vaccine for IAV-S would likely have been a more appropriate choice, but the only such commercially licensed product is no longer for purchase in the United States [47]. As development by animal health companies and universities into novel vaccine types continues, research and licensure studies should consider appropriate device configuration [48], such as the self-sprayer device, for delivery.

In contrast, live attenuated vaccines stimulate both systemic and mucosal immunity and can replicate locally at the administration site, making them more suitable for self-vaccination [28,29,30]. When paired with MP, the EE device has the potential to enhance vaccine uptake and immunogenicity by leveraging natural pig behavior in a timely, low-labor manner.

Live vaccines in contrast to killed vaccines tested here stimulated a more comprehensive immune response, including both systemic and mucosal immunity [28]. This dual response is particularly advantageous for respiratory or enteric pathogens, where mucosal immunity plays a crucial role [29,30]. Live attenuated and avirulent vaccine forms have the potential to replicate at the site of administration (such as the nasal or oral mucosa), which may lead to more robust local immunity. When delivered through the EE device, live vaccines could benefit from natural attraction induced by MP, as this encourages pigs to interact with the device, thereby increasing the likelihood of effective vaccine delivery and uptake.

Oral vaccines may be effective for self-administration as they stimulate mucosal immunity [49], essential for pathogens entering through the digestive or respiratory tracts, and the EE device combined with MP could facilitate their increased intake. In contrast, subunit and killed vaccines may be suitable due to a reliance on adjuvants, precise dosing, and a lack of mucosal stimulation, making them less effective for respiratory or mucosal pathogens in a self-administered format.

To measure oral fluid IgA, the commercial *M. hyopneumoniae* and *L. intracellularis* antibody ELISA kits were adapted into an indirect format. To our knowledge, this is the first report of the successful modification of the kit to measure IgA in oral fluids. Oral fluids are the most common sample type for surveillance and allow the sensitive screening of large populations at a lower cost and labor investment. In addition, the measurement of IgA suggests the ability of the immune system to neutralize the microorganism should infection occur.

Unfortunately, the Influenza ELISA adapted for the same purpose did not detect IgA antibody stimulated by the vaccine in oral fluid samples. Although, this adaptation has been successfully used to measure seroconversion following Influenza infection [19].

While the results of our study highlight the potential use of the EE device for effective immunization with attenuated vaccines, some limitations need to be acknowledged. Our study was conducted in a controlled research facility, which does not fully replicate the varied environmental conditions and operational challenges of commercial swine farms, where greater numbers of significantly more pigs are housed per pen. Importantly, we do not know how many pigs can be effectively vaccinated per device within a 6-h viability window of the vaccine, and this needs to be explored further in field study (for example, 25 pigs/sprayer). We also anticipate that this device will result in less variance at the pen level compared to traditional water-line delivery methods for these vaccines, making it a promising tool for large-scale operations.

Additionally, social hierarchy among pigs can influence access to the EE device, potentially affecting vaccine uptake in subordinate individuals. To mitigate this, future implementations in commercial settings likely require deploying multiple EE devices per pen as the inventory per pen increases to ensure equitable vaccine access across all social ranks. This strategy could help reduce variability in vaccine coverage within large groups.

Furthermore, age is a critical factor influencing vaccine efficacy, as younger pigs often exhibit more robust immune responses to vaccination. Implementing self-vaccination protocols at earlier stages could enhance immunological stability and contribute to the interruption of infection chains within herds. Appropriate vaccine timing varies by pathogen as well as age. Younger pigs often have maternal antibodies to IAV-S and *E. rhusiopathiae* that would necessitate administration at ages similar to those used in this study. Often, pigs are vaccinated out of convenience (i.e., at weaning time), not because this is the optimal timing of vaccination. Therefore, a delivery device for older pigs is desirable to deliver vaccines at a time when they are immunocompetent and seroconversion is not inhibited by maternal antibodies. Future studies should explore age-specific responses to optimize vaccination timing.

In summary, our study demonstrated the potential of the EE device with MP for administering attenuated vaccines to immunize pigs against endemic swine pathogens. The significant immune response in the Self-Vaccinated and Hand-Vaccinated groups for both the *ERY* (systemic and mucosal) and *LAW* (mucosal) vaccine trials highlighted the capability to enhance seroconversion-generated antibodies. These findings suggest that EE devices could significantly improve the precision of vaccination strategies, simplifying the process and reducing labor. Other vaccines are expected to work with this EE device if the formulation and method of delivery were adjusted. Self-vaccination is an advancement that could lead to improved animal welfare and productivity in commercial swine operations while saving labor.

## 5. Conclusions

EE devices or protocols are increasingly required by regulatory authorities and adopted by companies to enhance animal welfare and pig health. The integration of an EE device that reduces labor while improving vaccine delivery represents a significant advancement for swine health and welfare. The present study, along with previous findings, demonstrates that self-vaccination using an EE device is effective for Salmonella, *ERY*, and *LAW*. However, self-vaccination was not efficacious for killed IAV-S or *MHP* vaccines, likely due to limitations in vaccine antigen formulation for this delivery method.

Future improvements in vaccine formulations, such as the licensing of additional live attenuated vaccines or avirulent live cultures, or modifications to the EE device to facilitate intradermal (ID) or IM delivery, may enhance efficacy for IAV-S and *MHP* vaccines. Importantly, self-vaccination via an EE device not only reduces labor demands but also offers a less stressful and more enriching experience for pigs compared to traditional hand-vaccination methods. This approach has the potential to improve both vaccine compliance and overall animal welfare in commercial swine operations.

## 6. Patents

A patent was submitted and is pending by the corresponding author for the sprayer. The maternal pheromone patent (US12097181B2) was issued in 2024 to the corresponding author.

## Figures and Tables

**Table 1:** Experimental design of vaccine trials.

Trial	Vaccines	N	Sampling ^1^ Days Post-Vaccination
A	*MHP*	36	−7, 21, 28, 45
B	IAV	36	−7, 21, 28, 45
C	*ERY*	36	0, 21, 28, 42, 49
D	*LAW*	36	0, 21, 28, 42, 49

^1^ Sampling of oral fluids and blood serum.

**Table 2:** Antibody titer Least Squares Means ± SD in serum and oral fluids (data transformed log_10_(IgG + a)) for Trial A *MHP.*

	Treatment Groups			*p*-Value		
Measure	Control	Hand-Vaccinated	Self-Vaccinated	Time	Treatment ^c^	Treatment ∗ Time
***Serum***						
**Day −7, IgG**	0.012 ± 0.003 ^a^	0.032 ± 0.004 ^a^	0.019 ± 0.034 ^a^	0.206	<0.0001	<0.0001
**Day 21, IgG**	0.024 ± 0.004 ^a^	0.229 ± 0.022 ^b^	0.030 ± 0.005 ^a^			
**Day 28, IgG**	0.026 ± 0.004 ^a^	0.211 ± 0.020 ^b^	0.036 ± 0.006 ^a^			
**Day 45, IgG**	0.026 ± 0.003 ^a^	0.175 ± 0.016 ^b^	0.034 ± 0.005 ^a^			
***Oral Fluids***						
**Day −7, IgG**	0.083 ± 0.005 ^a^	0.007 ± 0.008 ^a^	0.081 ± 0.006 ^a^	<0.0001	<0.0001	<0.0001
**Day 21, IgG**	0.009 ± 0.008 ^a^	0.446 ± 0.050 ^b^	0.097 ± 0.008 ^a^			
**Day 28, IgG**	0.069 ± 0.006 ^a^	0.214 ± 0.024 ^b^	0.090 ± 0.009 ^a^			
**Day 45, IgG**	0.106 ± 0.046 ^a^	0.257 ± 0.044 ^b^	0.106 ± 0.008 ^a^			

^a,b^ The Least Square Means within a row with a different superscript differs significantly, and Tukey- Kramer post hoc test, *p* < 0.05. ^c^ Treatment effect was significant; however, the Hand-Vaccinated was higher post-vaccination than Control pigs while Self-Vaccinated pigs’ antibody titers did not differ from the Control values.

**Table 3:** Antibody titer to IAV-S are presented. Least Squares Means ± SEM in serum and oral fluids are shown in pigs in Trial B -IAV.

	Treatments			*p*-Value		
Measure	Control	Hand-Vaccinated	Self-Vaccinated	Treatment	Time	Treatment ∗ Time
***Serum***						
**Day −7, IgA**	0.153 ± 0.035 ^a^	0.083 ± 0.021 ^a^	0.225 ± 0.033 ^b^	0.200	0.001	0.009
**Day 21, IgA**	0.058 ± 0.029 ^a^	0.139 ± 0.035 ^b^	0.140 ± 0.038 ^b^			
**Day 28, IgA**	0.086 ± 0.025 ^a^	0.202 ± 0.037 ^b^	0.108 ± 0.002 ^a^			
**Day 45, IgA**	0.035 ± 0.042 ^a^	0.107 ± 0.034 ^a^	0.025 ± 0.028 ^a^			
**Day −7, TAB**	0.905 ± 0.023 ^a^	0.865 ± 0.046 ^a^	0.937 ± 0.027 ^a^	<0.0001	<0.0001	<0.0001
**Day 21, TAB**	0.835 ± 0.030 ^a^	0.761 ± 0.021 ^a^	0.793 ± 0.035 ^a^			
**Day 28, TAB**	0.851 ± 0.025 ^a^	0.395 ± 0.035 ^b^	0.915 ± 0.488 ^a^			
**Day 45, TAB**	0.916 ± 0.015 ^a^	0.499 ± 0.026 ^b^	0.882 ± 0.029 ^a^			
***Oral Fluids***						
**Day −7, IgA**	0.339 ± 0.094	0.097 ± 0.028	0.226 ± 0.054	0.543	0.139	0.056
**Day 21, IgA**	0.082 ± 0.048	0.159 ± 0.041	0.120 ± 0.045			
**Day 28, IgA**	0.100 ± 0.055	0.253 ± 0.043	0.266 ± 0.097			
**Day 45, IgA**	0.093 ± 0.028	0.246 ± 0.042	0.261 ± 0.135			

The Least Means Square within a row with a different superscript differs significantly; Tukey–Kramer post hoc test, *p* < 0.05.

**Table 4:** Antibody titer Least Mean Square ± SEM in serum (log_10_) and oral fluids (log_10_) for Trial C (*ERY*).

	Treatment			*p*-Value		
Measure	Control	Hand-Vaccinated	Self-Vaccinated	Time	Treatment	TIME ∗ Treatment
**Serum**						
**Day 0, IgG**	0.075 ± 0.014 ^a^	0.065 ± 0.016 ^a^	0.061 ± 0.020 ^a^	<0.0001	<0.0001	<0.0001
**Day 21, IgG**	0.004 ± 0.006 ^a^	0.578 ± 0.040 ^b^	0.684 ± 0.021 ^b^			
**Day 28, IgG**	0.037 ± 0.007 ^a^	0.573 ± 0.040 ^b^	0.709 ± 0.018 ^b^			
**Day 42, IgG**	0.041 ± 0.006 ^a^	0.544 ± 0.043 ^b^	0.655 ± 0.021 ^b^			
**Day 49, IgG**	0.039 ± 0.006 ^a^	0.542 ± 0.044 ^b^	0.709 ± 0.023 ^c^			
**Oral Fluids**						
**Day 0, IgG**	0.015 ± 0.003 ^a^	0.023 ± 0.009 ^a^	0.015 ± 0.003 ^a^	<0.0001	<0.0001	<0.0001
**Day 21, IgG**	0.008 ± 0.001 ^a^	0.324 ± 0.044 ^b^	0.625 ± 0.071 ^c^			
**Day 28, IgG**	0.013 ± 0.002 ^a^	0.496 ± 0.065 ^b^	0.474 ± 0.064 ^b^			
**Day 42, IgG**	0.014 ± 0.003 ^a^	0.457 ± 0.057 ^b^	0.518 ± 0.047 ^b^			
**Day 49, IgG**	0.022 ± 0.004 ^a^	0.422 ± 0.066 ^b^	0.556 ± 0.045 ^b^			

The Least Mean Square within a row with a different superscript differs significantly; Tukey–Kramer post hoc test, *p* < 0.05.

**Table 5:** Antibody titer Least Square Means ± SEM in serum and oral fluids for Trial D (*LAW*).

	Treatments			*p*-Value		
Measure	Control	Hand-Vaccinated	Self-Vaccinated	Treatment	Time	Treatment ∗ Time
***Serum***						
**Day 0, IgA**	1.404 ± 0.217	1.185 ± 0.208	1.178 ± 0.284	0.867	<0.0001	0.816
**Day 21, IgA**	0.580 ± 0.102	0.539 ± 0.082	0.518 ± 0.124			
**Day 28, IgA**	0.440 ± 0.093	0.503 ± 0.086	0.471 ± 0.121			
**Day 42, IgA**	0.475 ± 0.474	0.393 ± 0.047	0.440 ± 0.090			
**Day 49, IgA**	0.436 ± 0.436	0.286 ± 0.035	0.344 ± 0.064			
**Day 0, TAB**	72.224 ± 11.774	65.146 ± 16.528	63.012 ± 17.081	0.178	<0.0001	0.308
**Day 21, TAB**	59.570 ± 11.125	50.118 ± 15.932	53.115 ± 15.371			
**Day 28, TAB**	57.570 ± 11.784	47.637 ± 14.429	50.335 ± 16.108			
**Day 42, TAB**	55.303 ± 10.935	43.602 ± 12.183	49.015 ± 15.477			
**Day 49, TAB**	53.884 ± 12.228	40.632 ± 13.846	45.778 ± 15.573			
***Oral Fluids***						
**Day 0, IgA**	0.684 ± 0.119 ^a^	0.540 ± 0.083 ^a^	0.516 ± 0.105 ^a^	0.138	0.911	0.023
**Day 21, IgA**	0.326 ± 0.067 ^a^	0.623 ± 0.144 ^a,b^	0.823 ± 0.139 ^b^			
**Day 28, IgA**	0.336 ± 0.044 ^a^	0.730 ± 0.156 ^b^	0.898 ± 0.150 ^b^			
**Day 42, IgA**	0.482 ± 0.061 ^a^	0.680 ± 0.098 ^a^	0.617 ± 0.120 ^a^			
**Day 49, IgA**	0.612 ± 0.145 ^a^	0.637 ± 0.105 ^a^	0.522 ± 118 ^a^			

The Least Means Square within a row with a different superscript differs significantly; Tukey–Kramer post hoc test, *p* < 0.05.

## Data Availability

The data will be available in Supplementary Materials.

## Supplementary Materials

The following supporting information can be downloaded at: https://www.mdpi.com/article/10.3390/vaccines13030229/s1, Table S1: Detailed model selection criteria comparison, including Akaike Information Criterion (AIC), corrected Akaike Information Criterion (AICC), and Bayesian Information Criterion (BIC) before and after data transformation for IgG and IgA responses. Figure S1: Residual diagnostics for IgG oral fluids (MHP) before and after transformation. Panel (A) shows diagnostics before the transformation, with residuals exhibiting significant heteroscedasticity and deviation from normality. Panel (B) shows diagnostics after transformation, illustrating improved residual behavior. Figure S2: Residual diagnostics for IgG serum (MHP) before and after transformation. Panel (A) highlights variability before transformation, while Panel (B) demonstrates improvements in residual distribution and model assumptions after transformation. Figure S3: Residual diagnostics for IgA oral fluids (Erysipelas) before and after transformation. Residual variability and alignment with model assumptions improved post-transformation. Figure S4: Residual diagnostics for IgG serum (Erysipelas) before and after transformation. Transformation enhanced normality, reduced heteroscedasticity, and minimized outliers. Data Transformation Details: To address violations of statistical assumptions such as heteroscedasticity and non-normality, data were transformed using the formula: LY = log_10_(Y + a), where a = 1 − min(Y). This adjustment ensured all values were strictly positive, improving the statistical properties of the dataset while maintaining biological relevance. Detailed metrics for model selection criteria (AIC, AICC, and BIC) before and after transformation are provided in Table S1.

## References

[1] Selke M., Meens J., Springer S., Frank R., Gerlach G.-F. (2007). Immunization of Pigs To Prevent Disease in Humans: Construction and Protective Efficacy of a Salmonella Enterica Serovar Typhimurium Live Negative-Marker Vaccine. Infect. Immun..

[2] Boessen C., Artz G., Schulz L., Cook H. (2018). A Baseline Study of Labor Issues and Trends in U.S. Pork Production.

[3] Dubman R. (2021). Agricultural Income and Finance Situation and Outlook: 2021 Edition.

[4] Cardenas N.C., Valencio A., Sanchez F., O’Hara K.C. (2024). Analyzing the Intrastate and Interstate Swine Movement Network in the United States. Prev. Vet. Med..

[5] Chase C.C.L., Daniels C.S., Garcia R., Milward F., Nation T. (2008). Needle-Free Injection Technology in Swine: Progress toward Vaccine Efficacy and Pork Quality. J. Swine Health Prod..

[6] Mitragotri S. (2006). Current Status and Future Prospects of Needle-Free Liquid Jet Injectors. Nat. Rev. Drug Discov..

[7] Robbins R.C., Archer C., Giménez-Lirola L.G., Mora-Díaz J.C., McGlone J.J. (2023). Self-Administration of a Salmonella Vaccine by Domestic Pigs. Sci. Rep..

[8] McGlone J.J., Duke L., Sanchez M., Garcia A. (2023). Self-Administration of a Boar Priming Pheromone Stimulates Puberty in Gilts without Boar Exposure. Animals.

[9] Thacker E.L., Halbur P.G., Ross R.F., Thanawongnuwech R., Thacker B.J. (1999). Mycoplasma Hyopneumoniae Potentiation of Porcine Reproductive and Respiratory Syndrome Virus-Induced Pneumonia. J. Clin. Microbiol..

[10] Sørensen V., Ahrens P., Barfod K., Feenstra A.A., Feld N.C., Friis N.F., Bille-Hansen V., Jensen N.E., Pedersen M.W. (1997). Mycoplasma Hyopneumoniae Infection in Pigs: Duration of the Disease and Evaluation of Four Diagnostic Assays. Vet. Microbiol..

[11] Mancera Gracia J.C., Pearce D.S., Masic A., Balasch M. (2020). Influenza A Virus in Swine: Epidemiology, Challenges and Vaccination Strategies. Front. Vet. Sci..

[12] Do Nascimento G.M., Bugybayeva D., Patil V., Schrock J., Yadagiri G., Renukaradhya G.J., Diel D.G. (2023). An Orf-Virus (ORFV)-Based Vector Expressing a Consensus H1 Hemagglutinin Provides Protection against Di-verse Swine Influenza Viruses. Viruses.

[13] Bender J.S., Irwin C.K., Shen H.-G., Schwartz K.J., Opriessnig T. (2011). Erysipelothrix Spp. Genotypes, Serotypes, and Surface Protective Antigen Types Associated with Abattoir Condemnations. J. Vet. Diagn. Investig..

[14] Wang Q., Chang B.J., Riley T.V. (2010). Erysipelothrix Rhusiopathiae. Vet. Microbiol..

[15] Vannucci F.A., Gebhart C.J. (2014). Recent Advances in Understanding the Pathogenesis of Lawsonia Intracellularis Infections. Vet. Pathol..

[16] Tucker C.B., Mac-Neil M.D., Webster A.B. (2020). Guide for the Care and Use of Agricultural Animals in Research and Teaching.

[17] World Health Organization (WHO) (2014). WHO Policy Statement: Multi-Dose Vial Policy (MDVP) Revision 2014.

[18] Aviles-Rosa E.O., Surowiec K., McGlone J. (2020). Identification of Faecal Maternal Semiochemicals in Swine (Sus Scrofa) and Their Effects on Weaned Piglets. Sci. Rep..

[19] Panyasing Y., Goodell C.K., Giménez-Lirola L., Kittawornrat A., Wang C., Schwartz K.J., Zimmerman J.J. (2013). Kinetics of Influenza A Virus Nucleoprotein Antibody (IgM, IgA, and IgG) in Serum and Oral Fluid Specimens from Pigs Infected under Experimental Conditions. Vaccine.

[20] Giménez-Lirola L.G., Xiao C.T., Halbur P.G., Opriessnig T. (2012). Development of a Novel Fluorescent Microbead-Based Immunoassay and Comparison with Three Enzyme-Linked Immunoassays for Detection of Anti-Erysipelothrix Spp. IgG Antibodies in Pigs with Known and Unknown Exposure. J. Microbiol. Methods.

[21] Giménez-Lirola L.G., Xiao C.T., Halbur P.G., Opriessnig T. (2012). Development and Evaluation of an En-zyme-Linked Immunosorbent Assay Based on a Recombinant SpaA Protein (rSpaA415) for Detection of Anti-Erysipelothrix Spp. IgG Antibodies in Pigs. J. Microbiol. Methods.

[22] Xiao C.-T., Giménez-Lirola L.G., Gerber P.F., Jiang Y.-H., Halbur P.G., Opriessnig T. (2013). Identification and Characterization of Novel Porcine Astroviruses (PAstVs) with High Prevalence and Frequent Co-Infection of Individual Pigs with Multiple PAstV Types. J. Gen. Virol..

[23] Augustyniak A., Pomorska-Mól M. (2023). Vaccination failures in Pigs—The impact of chosen factors on the immunisation efficacy. Vaccines.

[24] Dumpa N., Goel K., Guo Y., McFall H., Pillai A.R., Shukla A., Repka M.A., Murthy S.N. (2019). Stability of Vaccines. AAPS PharmSciTech.

[25] Schmied J., Hamilton K., Rupa P., Oh S.-Y., Wilkie B. (2012). Immune Response Phenotype Induced by Controlled Immunization of Neonatal Pigs Varies in Type 1:Type 2 Bias. Vet. Immunol. Immunopathol..

[26] Betlach A. (2021). Approaches for Mycoplasma Hyopneumoniae Detection, Control, and Molecular Characterization. Ph.D. Thesis.

[27] Feng Z.-X., Wei Y.-N., Li G.-L., Lu X.-M., Wan X.-F., Pharr G.T., Wang Z.-W., Kong M., Gan Y., Bai F.-F. (2013). Development and Validation of an Attenuated Mycoplasma Hyopneumoniae Aerosol Vaccine. Vet. Microbiol..

[28] Seo S.-U., Seong B.-L. (2022). Prospects on Repurposing a Live Attenuated Vaccine for the Control of Unrelated Infections. Front. Immunol..

[29] Perez-Lopez A., Behnsen J., Nuccio S.-P., Raffatellu M. (2016). Mucosal Immunity to Pathogenic Intestinal Bacteria. Nat. Rev. Immunol..

[30] Park S.-C., Wiest M.J., Yan V., Wong P.T., Schotsaert M. (2024). Induction of Protective Immune Responses at Respiratory Mucosal Sites. Hum. Vaccines Immunother..

[31] Maes D., Sibila M., Kuhnert P., Segalés J., Haesebrouck F., Pieters M. (2018). Update on Mycoplasma Hyopneumoniae Infections in Pigs: Knowledge Gaps for Improved Disease Control. Transbound. Emerg. Dis..

[32] Larsen D.L., Karasin A., Zuckermann F., Olsen C.W. (2000). Systemic and Mucosal Immune Responses to H1N1 Influenza Virus Infection in Pigs. Vet. Microbiol..

[33] Keshavarz M., Mirzaei H., Salemi M., Momeni F., Javad Mousavi M., Sadeghalvad M., Arjeini Y., Solay-mani-Mohammadi F., Sadri Nahand J., Namdari H. (2018). Influenza Vaccine: Where Are We and Where Do We Go?. Med. Virol..

[34] Krammer F. (2019). The human antibody response to influenza A virus infection and vaccination. Nat. Rev. Immunol..

[35] Hajra D., Datey A., Chakravortty D. (2020). Attenuation methods for live vaccines. Methods Mol. Biol..

[36] Graaf-Rau A., Schmies K., Breithaupt A., Ciminski K., Zimmer G., Summerfield A., Sehl-Ewert J., Lillie-Jaschniski K., Helmer C., Bielenberg W. (2024). Reassortment incompetent live attenuated and replicon influenza vaccines provide improved protection against influenza in piglets. NPJ Vaccines.

[37] Ogra P.L., Faden H., Welliver R.C. (2001). Vaccination Strategies for Mucosal Immune Responses. Clin. Microbiol. Rev..

[38] Brandtzaeg P. (2013). Secretory IgA: Designed for Anti-Microbial Defense. Front. Immunol..

[39] Guedes R.M.C., Gebhart C.J. (2010). Evidence of Cell-Mediated Immune Response and Specific Local Mucosal Immunoglobulin (Ig) A Production against Lawsonia Intracellularis in Experimentally Infected Swine. Can. J. Vet. Res..

[40] Sattler K., Billing M., Gimenez-Lirola L., Magtoto R., Mora-Diaz J., Leite F. (2023). Evaluation of the IgA Response to Attenuated-Live Oral Lawsonia Intracellularis Vaccine. Proceedings of the AASV Annual Meeting.

[41] Maltseva M., Galipeau Y., Renner T.M., Deschatelets L., Durocher Y., Akache B., Langlois M.-A. (2022). Characterization of Systemic and Mucosal Humoral Immune Responses to an Adjuvanted Intranasal SARS-CoV-2 Protein Subunit Vaccine Candidate in Mice. Vaccines.

[42] Chen D., Periwal S.B., Larrivee K., Zuleger C., Erickson C.A., Endres R.L., Payne L.G. (2001). Serum and Mucosal Immune Responses to an Inactivated Influenza Virus Vaccine Induced by Epidermal Powder Immunization. J. Virol..

[43] Schmader K.E., Liu C.K., Flannery B., Rountree W., Auerbach H., Barnett E.D., Schlaudecker E.P., Todd C.A., Poniewierski M., Staat M.A. (2023). Immunogenicity of Adjuvanted versus High-Dose Inactivated Influenza Vaccines in Older Adults: A Randomized Clinical Trial. Immun. Ageing.

[44] Zhao T., Cai Y., Jiang Y., He X., Wei Y., Yu Y., Tian X. (2023). Vaccine Adjuvants: Mechanisms and Platforms. Sig. Transduct. Target. Ther..

[45] Han S., Lee P., Choi H. (2023). Non-Invasive vaccines: Challenges in formulation and vaccine adjuvants. Pharmaceutics.

[46] Kamboj A., Dumka S., Saxena M.K., Singh Y., Kaur B.P., Da Silva S.J.R., Kumar S. (2024). A comprehensive review of our understanding and challenges of viral vaccines against swine pathogens. Viruses.

[47] Petro-Turnquist E., Pekarek M.J., Weaver E.A. (2024). Swine influenza A virus: Challenges and novel vaccine strategies. Front. Cell Infect. Microbiol..

[48] Wu L., Xu W., Jiang H., Yang M., Cun D. (2024). Respiratory delivered vaccines: Current status and perspectives in rational formulation design. Acta Pharm. Sin. B.

[49] Lavelle E.C., Ward R.W. (2022). Mucosal Vaccines—Fortifying the Frontiers. Nat. Rev. Immunol..

