# Supplementary material for: Efficacy of a Self-Vaccination Strategy for Influenza A Virus, Mycoplasma hyopneumoniae, Erysipelothrix rhusiopathiae, and Lawsonia intracellularis in Swine
Source: Vaccines (Basel). 2025 Feb 24;13(3):229. doi: 10.3390/vaccines13030229 (PMC11946863; doi:10.3390/vaccines13030229)
Supplement: Supplementary file 1 [file vaccines-13-00229-s001.zip › Supplemental materials -- data transformations.pdf]

Innovative Self-Vaccination Strategy for Swine Influenza A virus, *Mycoplasma hyopneumoniae, Erysipelothrix rhusiopathiae*, and *Lawsonia intracellularis* - Data Transformation

Transforming data is a fundamental technique in exploratory data analysis, with centering and scaling being basic examples. More complex transformations, like log transformation, are invaluable for addressing violations of statistical assumptions. For instance, when a variable is not normally distributed, a log transformation can help improve normality and fix the heteroscedasticity. This transformation is especially effective for variables that are strictly positive, making it widely applicable in biological and other scientific research. Even when the data includes small or negative values, adjustment can be made to apply the log transformation effectively, ensuring it is relevance across diverse datasets. In our case, the data originated from ELISA immunoassay, where some values were negative because the samples were compared to a control sample. To apply the log transformation effectively, adjustments were made to ensure all values were positive, allowing to improve the statistical properties of the dataset while maintaining biological relevance.

A common practice for handling negative values is to add constant value to the data to applying the log transformation (Sokal and Rohlf, 1995). Building on this approach, we utilized a transformation formula that adjusts for negative values by adding a constant before applying the log transformation. Specifically, the minimum value in the dataset was identified and incorporated in the formula as $\mathbf{LY = Log_{10}(Y + a)}$, where $\mathbf{a = 1 - min(Y)}$. This method not only shifted the entire dataset to a strictly positive range but also preserved the relative differences between observations. This strategy aligns with established practices in statistical transformation and is particularly relevant dataset where values are derived from comparative assays.

To evaluate the suitability and fit of the mixed-effect model applied to the transformed data, model select criteria such as Akaike Information Criterion (AIC), corrected Akaike Information Criterion (AICC), Bayesian Information Criterion (BIC) were used. These metrics allowed for comparison between comparing models and provided insights into the balance between model complexity and goodness-of-fit. A model with lower AIC, AICC, and BIC values was considered preferable ensuring that the transformation and statistical approach yield a robust and parsimonious model suitable for reliable interpretation of the results.

Results

The transformation applied to the Mycoplasma IgG oral fluids significantly improved the model fit, as evidence by reductions in key model selection criteria values, specifically after the transformation, the AIC decreased from 149.71 to -227.21, the AICC decreased from 144.91 to -277.01, and BIC decreased from 149.38 TO -272.54, details for residuals normality and equal variance can be checked on figure 1. For Mycoplasma IgG serum, the transformation resulted in a decrease in AIC from -201.30 to -527.99, AICC from -201.21 to -527.89, and BIC from -198.14 to -524.82, details for residuals normality and equal variance can be checked on figure 2.

Similarly, for Erysipelas IgA oral fluids, the AIC decreased from 542.71 to -137.10, the AICC from 542.86 to -136.95, and the BIC from 547.46 to -132.35, details for residuals normality and equal variance can be checked on figure 3. For Erysipelas IgG serum, the AIC decreased drastically from 290.47 to -409.33, the AICC from 290.45 to -409.26, and the BIC from 293.63 to -406.16, details for residuals normality and equal variance can be checked on figure 3. These reductions indicate that the transformed models are not only more parsimonious but also better suited to the data, improving the ability to explain variability while minimizing complexity. The detailed values for AIC, AICC, and BIC before and after transformation are provided in Table 1. This highlights the effectiveness of the transformation in optimizing the models' performance.

Table 1. Detailed model selection criteria comparison

Model	AIC (Before)	AIC (After)	AICC (Before)	AICC(After)	BIC (Before)	BIC (After)
**IgG OF -MhP**	144.71	-277.21	144.91	-277.01	149.38	-272.54
**IgG Serum - MhP**	-201.30	-527.99	-201.21	-527.89	-198.14	-524.82
**IgA OF- Ery**	542.71	-131.10	542.86	-136.95	547.46	-131.35
**IgG Serum - Ery**	290.47	-409.33	290.45	-409.26	293.63	-406.16

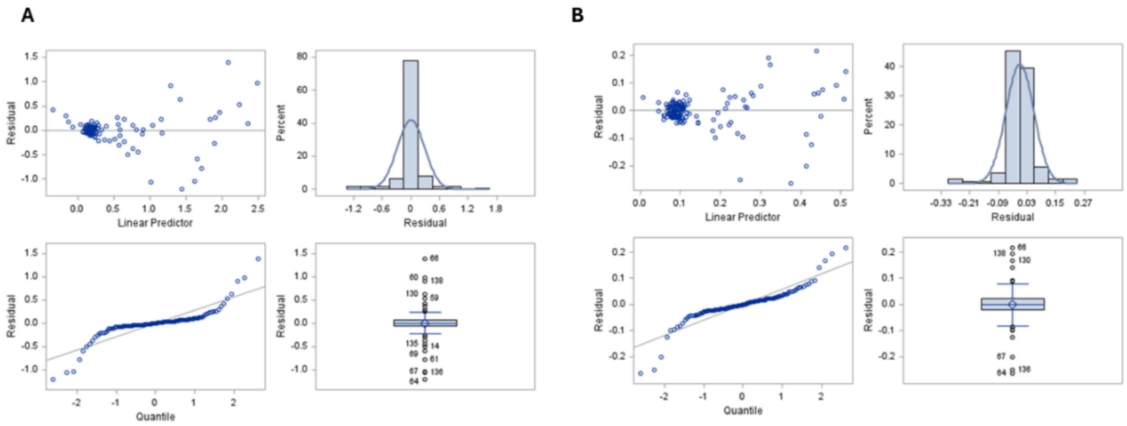

Figure 1. Residual diagnostics for IgG oral fluids mycoplasma before and after data transformation. Panel (A) shows residual diagnostics before the log transformation, while Panel (B) illustrates the same diagnostics after transformation. The Residual vs. Linear Predictor plots (top-left) demonstrate reduced heteroscedasticity in Panel (B), as residuals are more evenly distributed around zero. The histograms of residuals (top-right) show improved normality in Panel (B), with residuals closely following the overlaid normal curve. The Q-Q plots (bottom-left) confirm better alignment with the theoretical quantiles of a normal distribution post-transformation. Finally, the boxplots (bottom-right) highlight a more compact distribution of residuals and fewer outliers in Panel (B). These

improvements underscore the effectiveness of data transformation in meeting model assumptions and enhancing the reliability of the analysis.

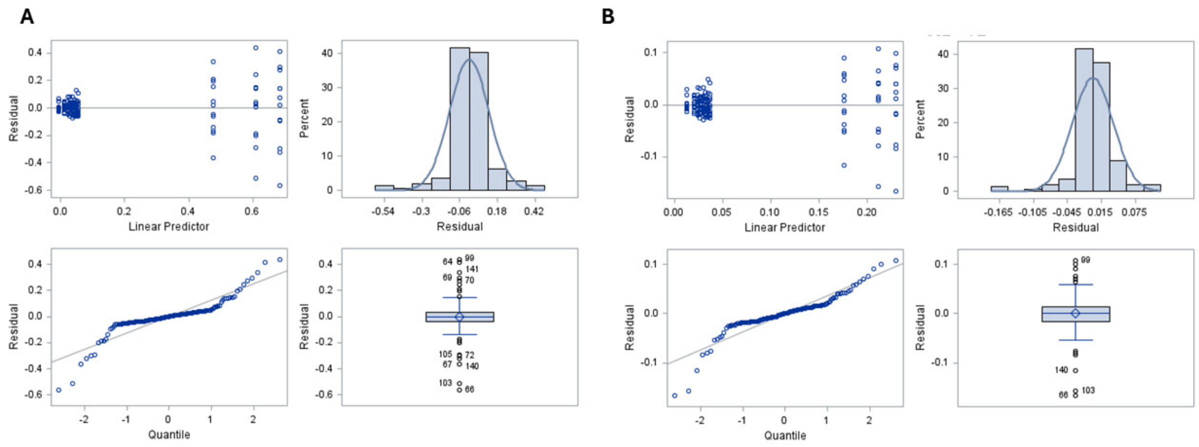

Figure 2. Residual diagnostics for IgG serum mycoplasma before and after data transformation. Panel (A) shows residual diagnostics before the log transformation, while Panel (B) illustrates the same diagnostics after transformation. The Residual vs. Linear Predictor plots (top-left) demonstrate reduced heteroscedasticity in Panel (B), as residuals are more evenly distributed around zero. The histograms of residuals (top-right) show improved normality in Panel (B), with residuals closely following the overlaid normal curve. The Q-Q plots (bottom-left) confirm better alignment with the theoretical quantiles of a normal distribution post-transformation. Finally, the boxplots (bottom-right) highlight a more compact distribution of residuals and fewer outliers in Panel (B). These improvements underscore the effectiveness of data transformation in meeting model assumptions and enhancing the reliability of the analysis.

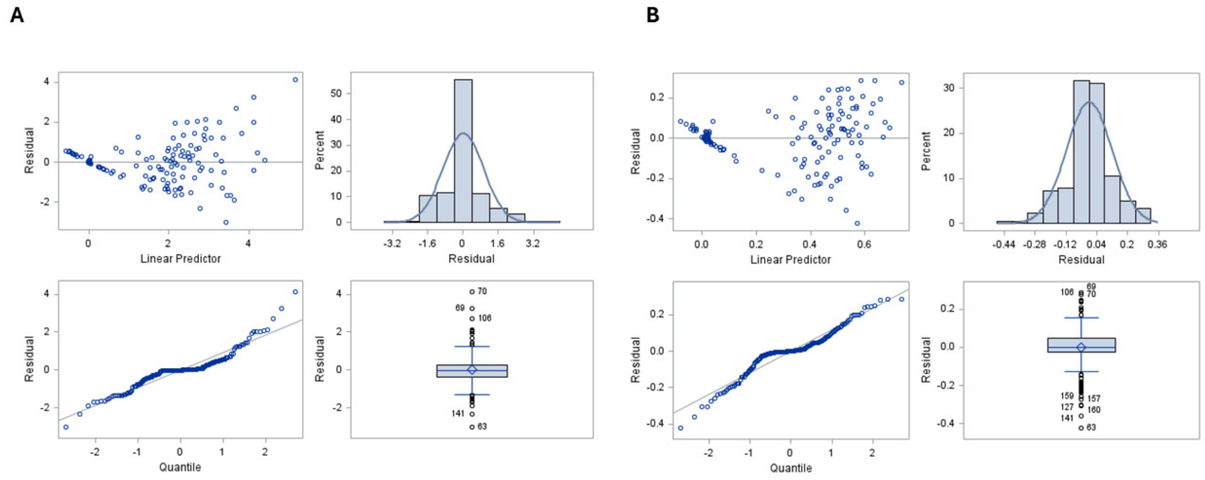

Figure 3. Residual diagnostics for IgA oral fluids Erysipelas before and after data transformation. Panel (A) shows residual diagnostics before the log transformation, while Panel (B) illustrates the same diagnostics after transformation. The Residual vs. Linear Predictor plots (top-left) demonstrate reduced heteroscedasticity in Panel (B), as residuals are more evenly distributed around zero. The histograms of residuals (top-right) show improved normality in Panel (B), with residuals closely following the overlaid normal curve. The Q-Q plots (bottom-left) confirm better alignment with the theoretical quantiles of a normal distribution post-transformation. Finally, the boxplots (bottom-right) highlight a more compact distribution of residuals and fewer outliers in Panel (B). These improvements underscore the effectiveness of data transformation in meeting model assumptions and enhancing the reliability of the analysis.

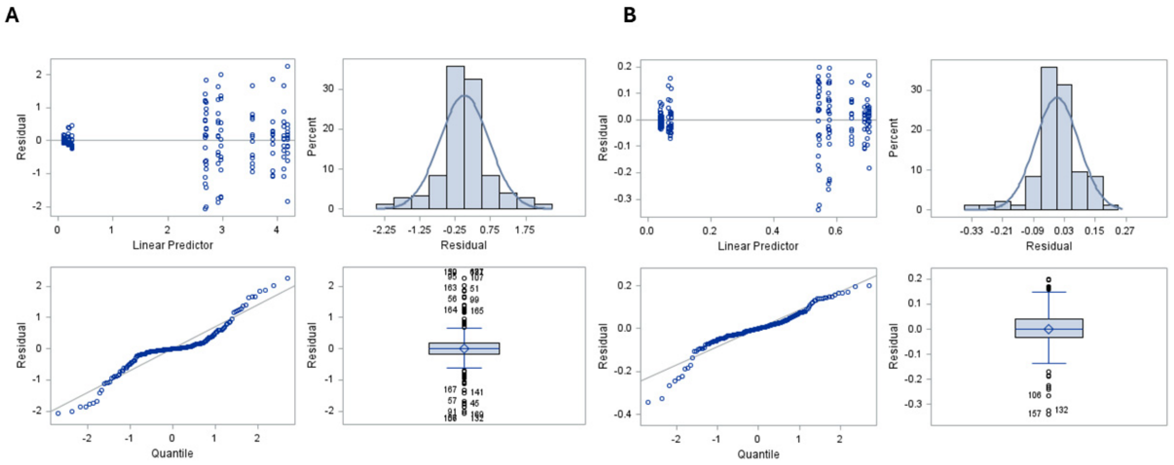

Figure 4. Residual diagnostics for IgG serum Erysipelas before and after data transformation. Panel (A) shows residual diagnostics before the log transformation, while Panel (B) illustrates the same diagnostics after transformation. The Residual vs. Linear Predictor plots (top-left) demonstrate reduced heteroscedasticity in Panel (B), as residuals are more evenly distributed around zero. The histograms of residuals (top-right) show improved normality in Panel (B), with residuals closely following the overlaid normal curve. The Q-Q plots (bottom-left) confirm better alignment with the theoretical quantiles of a normal distribution post-transformation. Finally, the boxplots (bottom-right) highlight a more compact distribution of residuals and fewer outliers in Panel (B). These improvements underscore the effectiveness of data transformation in meeting model assumptions and enhancing the reliability of the analysis.
