# Supplementary figures and images for: Efficacy of a Self-Vaccination Strategy for Influenza A Virus, Mycoplasma hyopneumoniae, Erysipelothrix rhusiopathiae, and Lawsonia intracellularis in Swine
Source: Vaccines (Basel). 2025 Feb 24;13(3):229. doi: 10.3390/vaccines13030229 (PMC11946863; doi:10.3390/vaccines13030229)

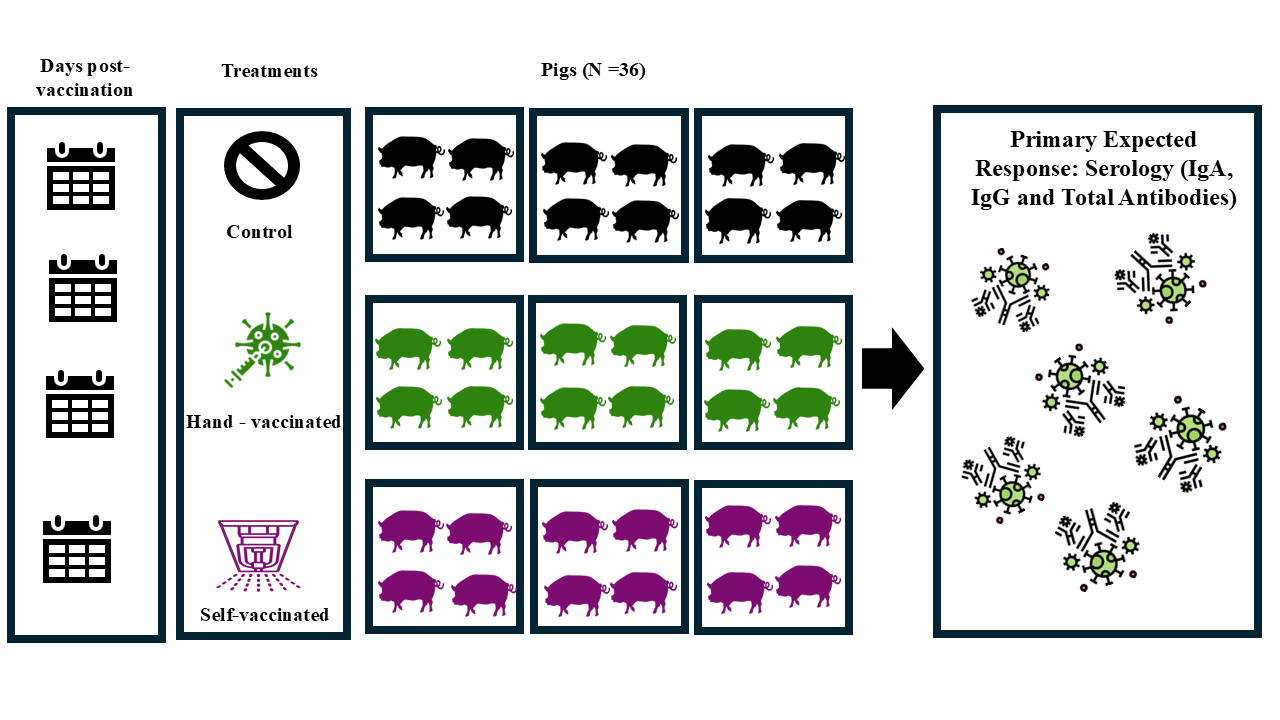

Supplement: Supplementary file 1 [file vaccines-13-00229-s001.zip › Experimental Design - Self-Vacination Pigs.png]
